# Immunophenotyping of Paired Bone Marrow and Peripheral Blood: Implications for Monitoring Plasma Cell Disorders

**DOI:** 10.3390/cancers18213428

**Published:** 2026-10-23

**Authors:** Janine M. Joseph, Joseph D. Tario, Han Yu, Sarah Parker, Ian Lund, Kristina Slemmer, Megan E. Schaefer, Kimberly L. Celotto, Hamza Hassan, Philip L. McCarthy, Hemn Mohammadpour, Rikki Cannioto, Jens Hillengass

**Affiliations:** 1 Department of Cancer Prevention and Control, Roswell Park Comprehensive Cancer Center, Elm & Carlton Streets, Buffalo, NY 14263, USA; rikki.cannioto@roswellpark.org; 2 Flow Cytometry Laboratory (Clinical), Roswell Park Comprehensive Cancer Center, Buffalo, NY 14263, USA; joseph.tario@roswellpark.org; 3 Department of Biostatistics and Bioinformatics, Roswell Park Comprehensive Cancer Center, Buffalo, NY 14263, USA; han.yu@roswellpark.org; 4 Department of Medicine, Roswell Park Comprehensive Cancer Center, Buffalo, NY 14263, USA; sarah.parker@roswellpark.org (S.P.); ian.lund@roswellpark.org (I.L.); kristina.slemmer@roswellpark.org (K.S.); megan.schaefer@roswellpark.org (M.E.S.); kimberly.celotto@roswellpark.org (K.L.C.); hamza.hassan@roswellpark.org (H.H.); philip.mccarthy@roswellpark.org (P.L.M.); jens.hillengass@roswellpark.org (J.H.); 5 Department of Cell Stress Biology, Roswell Park Comprehensive Cancer Center, Buffalo, NY 14263, USA; hemn.mohammadpour@roswellpark.org

**Keywords:** plasma cell disorders, immunophenotyping, flow cytometry, bone marrow, peripheral blood, paired samples

## Abstract

Background: Although peripheral blood (PB) immunophenotyping is used to monitor immune reconstitution after transplant, there is almost no understanding of how closely the PB is correlated with the bone marrow (BM)—the tumor immune microenvironment (TIME)—in plasma cell disorders (PCD). The invasiveness of BM biopsies and spatial heterogeneity of cancer infiltration limit their use. Methods: To address this gap in knowledge, paired pre-treatment PB and BM aspirate (BMA) samples from 60 patients with PCDs were immunophenotyped with multicolor flow cytometry. Thirty lymphocyte populations and 11 ratios were quantified for each compartment. Abundance was expressed as median and BMA-PB correlation as Spearman’s correlation (ρ). Results: The largest differences in abundance are in CD8^+^ T cells: effector memory (+26% in BMA); and cells co-expressing ≥ 2 exhaustion-associated markers (+33% in BMA). Despite differential abundance, the BMA and PB of all markers are significantly correlated, with almost all having very strong or strong correlations, the strongest being the cytotoxic populations gamma-delta T cells (ρ = 0.95), CD8^+^ T cells (ρ = 0.96), and CD57^+^ NKT (ρ = 0.97). Exploratory stratified analyses revealed that most correlations remained strong even in subgroups defined by age, sex, obesity, symptom burden, purity of BMA sample, and days between PB and BMA sample. An illustrative single-patient exploratory analysis reveals that the focal lesion immune composition is closer to the PB than BMA. Conclusions: This study suggests that the PB provides insight into the PCD TIME, and further evaluation of PB immunophenotyping as a less-invasive approach for estimating selected BMA immune populations is warranted.

## Simple Summary

Patients being evaluated for multiple myeloma must undergo a bone marrow biopsy for definitive diagnosis. Moreover, bone marrow biopsies are required to monitor response to treatment and immune reconstitution after transplant and to investigate suspected relapse. These procedures provide critical information about disease burden and prognostic characteristics but not about the overall immune profile of the bone marrow. Moreover, the procedures are quite unpleasant for patients. The current analysis was performed to examine how closely immune cell populations and key ratios of populations are correlated in the bone marrow aspirate and peripheral blood—a more readily accessible biospecimen. Most markers examined have a strong statistical correlation in the two compartments, and several populations, including the very important non-exhausted cytotoxic (CD8^+^) T cells, have both a very similar abundance and very strong correlation, suggesting that the peripheral blood should be further explored as a possible surrogate for the bone marrow for several key immune cell populations.

## 1. Background

### 1.1. Multiple Myeloma

Multiple myeloma (MM) is a hematologic malignancy in which dysfunctional plasma cells (PC) infiltrate the bone marrow (BM), oftentimes leading to immunoparesis or CRAB features (hypercalcemia, renal failure, anemia, bone lesions). It is the second most common hematologic malignancy in developed countries [1]. In 2026 in the United States, there will be an estimated 36,000 new cases of MM and 10,850 deaths [2]. In the past 20 years, the five-year relative survival has nearly doubled [2], due in large part to novel treatments and combinations [3].

### 1.2. Diagnosis of MM

The initial diagnostic workup for MM includes imaging (magnetic resonance imaging, whole-body computed tomography, or positron emission tomography/computed tomography), blood and urine tests (complete blood count, serum calcium, serum creatinine, serum and urine protein electrophoresis, and free light chain assays), and BM biopsy to detect malignant PCs, to quantify the disease burden, and to evaluate cytogenetic profile—but not to conduct detailed immunophenotyping of the tumor immune microenvironment (TIME) [4]. Imaging and biopsy aid in the characterization of the infiltration pattern in the BM, which manifests most frequently as focal tumors of malignant PCs. However, a small proportion of patients present with homogenous diffuse infiltration or a combination of both patterns.

### 1.3. Role of the Immune System in MM Treatment

Certain treatments for MM rely on a well-functioning immune system capable of responding in a sufficient and predictable manner. Immunotherapies work by targeting the immune suppression in the BM TIME and boosting the innate and adaptive immune responses. Immunomodulatory drugs (IMiD) and monoclonal antibodies work by activating natural killer (NK) cells, gamma-delta (γδ) T cells, CD4^+^ T cells, CD8^+^ T cells, and macrophages and by suppressing myeloid-derived suppressor cells and regulatory T (Treg) and B (Breg) cells [5]. Chimeric antigen receptor (CAR) T cells rely on the patient’s capacity to promote the persistence, expansion, and recruitment of other immune cells. If the TIME is too immunosuppressive, the CAR T cells can become exhausted [6]. The chronic activation of T cells caused by bispecific T cell engagers can lead to T cell exhaustion [7,8].

### 1.4. Immune Profile Association with Response to Treatment

Certain immune characteristics have been associated with better clinical outcomes in patients with plasma cell disorders (PCD), including BM immune reactivity and post-therapy immune normalization in high-risk smoldering multiple myeloma (HRSMM) [9], NK cell levels in the BM in MM patients receiving autologous stem cell transplant [10], and, in newly diagnosed MM, naïve B-cell abundance [11], lower levels of exhausted and senescent T cells in the BM, and lower markers of subsets suggestive of older immune age, including activated, differentiated, and senescent T cells in the BM and peripheral blood (PB) [12,13]. In relapsed/refractory MM, better clinical outcomes have been associated with lower levels of exhausted and (sustained) activated T cells, Tregs, and γδ T cells and higher cytotoxic T cell infiltration in the BM, and lower levels of Tregs and T cell exhaustion markers and higher levels of T cells, cytolytic T cells, and (transiently) activated T cells in the PB [14,15,16].

### 1.5. Heterogeneity of BM Infiltration

Imaging reveals that myeloma infiltrates the BM in different patterns, meaning that a BM biopsy can provide an incomplete picture of the TIME. The patterns within the BM can be diffuse and homogeneous but also present as focal lesions—small PC tumors inside or outside the BM space [17]. Biopsies of focal lesions have shown that not only are the malignant PCs genomically different in focal lesions compared to the diffusely infiltrated BM but that the TIME also shows a more inflammatory immunosuppressive environment in focal lesions [18,19,20,21]. Biopsies of different areas of the body in one setting are time-consuming and unpleasant for the patient, making the PB an attractive candidate to explore as a surrogate for the BM to be used in the diagnosis and monitoring of PCDs.

The BM and PB share some similarities, due, in part, to the frequent movement of T cells between the PB and BM, but there are differences between these two compartments, which are important to explore to elucidate the potential for PB as a surrogate for BM biopsy. Given the importance of BM immune profile dynamics in PCD outcomes, the difficulty of sampling BM relative to PB, the spatial heterogeneity inherent to the BM in MM, and the possibility of contamination of BM biopsy samples with blood, further exploration of the extent to which the PB immune profile reflects that of the BM is warranted.

## 2. Methods

### 2.1. Study Population

Patients considered for inclusion in this retrospective cross-sectional hospital-based study were being evaluated for PCDs at Roswell Park Comprehensive Cancer Center in Buffalo, New York, USA (Roswell Park) between 2020 and 2024. Patients were included if they were treatment naïve at the time of both PB and BM aspirate (BMA) sampling, had a pre-treatment PB draw and BM biopsy within 15 days of one another, and had immunophenotyping performed with the identical flow cytometry panel on both PB and BMA. The study was approved by the Roswell Park Institutional Review Board (BDR-131920).

### 2.2. Biospecimen Collection and Processing

BMA and PB samples are collected as part of the clinical routine into sodium heparin vacutainer tubes in the Roswell Park Myeloma Clinic and central phlebotomy, respectively, as part of the standard-of-care for patients being worked up for PCDs. The order of the BMA (first-pull vs. later aspirates) is not explicitly documented. Once collected, specimens are sent to the Clinical Flow Cytometry Laboratory, where they are accessioned and held at room temperature prior to and during processing. Processing generally occurs the same day. Laboratory quality control studies have shown that these samples are stable under these conditions for up to 48 h. An aliquot is removed for immunophenotyping [22], and RPMI 1640-based holding medium is added to the remnant material, which is held at 2–8 °C for approximately one week and subsequently discarded if not needed.

### 2.3. Immunophenotyping

Five eight-color immunophenotyping panels are evaluated, the purposes of which are to discriminate and quantify major lymphocyte populations and T, B, and NK cell subsets. Table S1 contains descriptions of the cell surface markers used to characterize each population. The gating strategy and reagents used for each cell surface marker are described in the Supplementary Materials, Files S1 and S2.

The current analysis includes the following lymphocyte populations: total lymphocytes, total T lymphocytes (T cells), γδ T cells, CD4^+^ and CD8^+^ T cells and functional subsets, including naïve, central memory (TCM), effector memory (TEM), terminally differentiated TEM/TEM re-expressing CD45RA (TEMRA), and Treg, non-exhausted, and exhausted. Exhausted T cells within each major T cell lineage (CD4^+^, CD8^+^) are defined as co-expressing two or more of the following cell surface markers: PD-1, TIGIT, TIM-3, LAG-3; non-exhausted T cells are defined as expressing none of those four markers. The analysis also includes total B lymphocytes (B cells) and functional subsets, including naïve, total memory, non-class-switched memory (NCSM), and class-switched memory (CSM). Lastly, the analysis includes both CD57^+^ and CD57^−^ NK and NKT cells, and CD57^+^ large granular lymphocytes (LGLs).

For the purposes of data analysis, populations are expressed as a percentage of the parent gate (percent gated [PG]), with lymphocytes expressed as a percentage of total leukocytes and all other sub-populations expressed as a percentage of total lymphocytes. Ratios of sub-populations are calculated to highlight functional relationships, including CD4^+^/CD8^+^ T cells, CD4^+^ Tregs/CD8^+^ T cells, CD4^+^ naïve/memory, CD4^+^ TEM/TCM, CD4^+^ exhausted/non-exhausted, CD4^+^ TEM/exhausted, CD8^+^ naïve/memory, CD8^+^ TEM/TCM, CD8^+^ exhausted/non-exhausted, CD8^+^ TEM/exhausted, and B cells naïve/memory. In total, data are reported for 30 populations and 11 ratios, collectively referred to as the 41 “markers”.

### 2.4. Statistical Methods

To examine both agreement and statistical correlation, the BMA and PB are compared on two criteria: (1) absolute quantification of immune markers in the two compartments and (2) the statistical correlation between the two compartments’ markers, irrespective of differences in population size. For each of the 41 markers, median values and interquartile ranges are reported for the BMA and PB compartments. Study sample median differences between the BMA and PB compartments are stated as a percentage of the PB median value and tested for significance using the Wilcoxon signed-rank test. Correlations between paired BMA and PB are reported using Spearman’s rank correlation coefficients (ρ) and 95% confidence intervals (CI). The strength of each Spearman correlation is classified thus: |ρ| ≥ 0.90: very strong; 0.70 ≤ |ρ| < 0.90: strong; 0.50 ≤ |ρ| < 0.70: moderate; 0.30 ≤ |ρ| < 0.50: weak; |ρ| < 0.30: negligible. *p*-values are corrected for the false discovery rate (FDR) using the Benjamini–Hochberg (BH) procedure.

Principal components analysis (PCA) was performed on the immune markers, excluding the ratios. Multivariate outliers were identified using Mahalanobis distance in the first two PCA dimensions, with samples exceeding the 99th percentile of the chi-square distribution excluded from subsequent analyses. Partial least squares discriminant analysis (PLS-DA) was then performed on the resulting dataset with unit-variance scaling. The top 25% of markers contributing to the first component were visualized in a heatmap based on loading weights. For heatmap visualization, biomarker abundances were Winsorized at the 5th and 95th percentiles and standardized. Hierarchical clustering was performed using complete linkage.

### 2.5. Exploratory Analysis of Correlations by Subgroup

Exploratory subgroup analyses are used to generate hypotheses regarding the effect that patient or disease characteristics have on the observed relationships between the paired BMA and PB correlations, using the same methodology to report and test correlations within each subgroup as in the combined analysis. A bootstrap-based test with 1000 iterations is used to compare the correlation coefficients of the two subgroups, with the 95% CI for difference in ρ between the subgroups reported. A CI that does not include 0 is indicative of a statistically significant difference in correlation between the two subgroups. The subgroups examined include sex (male vs. female); obesity status (obese vs. non-obese); time between BMA and PB sampling (1 day vs. >1 day); diagnosis (asymptomatic [MGUS, SMM, negative for all, not definitive] vs. symptomatic [MM, plasmacytoma, AL amyloidosis, or plasma cell leukemia]); age (40–65 vs. >65 years); and BMA cellularity (>10,500 vs. ≤10,500 leukocytes per microliter [μL]). The final subgroup analysis uses ≤10,500 leukocytes per μL as an approximate threshold to establish BMA samples which are presumably heavily contaminated by blood, given the PB leukocyte reference range of 3500 to 10,500 cells per μL in older adults with MM [23].

### 2.6. Exploratory Analysis of Paired Lesion, BMA, and PB

Exploratory analysis was conducted on one patient who had immunophenotyping data on PB and both focal lesion and BM biopsies, with the BM and focal lesion biopsies performed on the same day, eight days after the PB draw. Because there are data available from only one patient, the analysis is restricted to the major populations—T cells, CD4^+^ T cells, CD8^+^ T cells, exhausted and non-exhausted subsets, B cells, NK cells, and NKT cells—as a percentage of total lymphocytes in each of the three compartments. The PB value and BMA value are each stated as a percentage of the lesion value, with values close to 100% suggesting relatively close concordance between the PB/lesion or BMA/lesion.

Analyses were conducted in SAS 9.4 for Windows (Cary, NC, USA) and R/RStudio 2026.01.1 Build 403 (Posit Software, PBC).

## 3. Results

### 3.1. Patient Characteristics

As shown in Table 1, patients in the study (*n* = 60) have a mean (standard deviation [SD]) age of 66.5 (9.9) years at the time of immunophenotyping. The PB and BMA samples were taken 0–15 (mean 3.8) days apart. The individuals are generally overweight, with a mean (SD) body mass index of 29.8 (5.9) kg/m^2^. The majority are male (68.3%) and non-Hispanic White (75.0%). Only 3.3% are current smokers. More than half of the individuals have treatment-naïve MM (60.0%), and more than one-quarter have an MM precursor condition, including MGUS (18.3%) and SMM (8.3%). Patients with MM are more likely to be non-Hispanic White, have a light-chain subtype, and as expected, have higher bone marrow PC percentage and serum monoclonal (M) protein compared to those with other conditions. The majority (83.3%) of patients with MM have one or more CRAB features.

### 3.2. Immune Marker Abundance by Compartment

**Figure 1:**
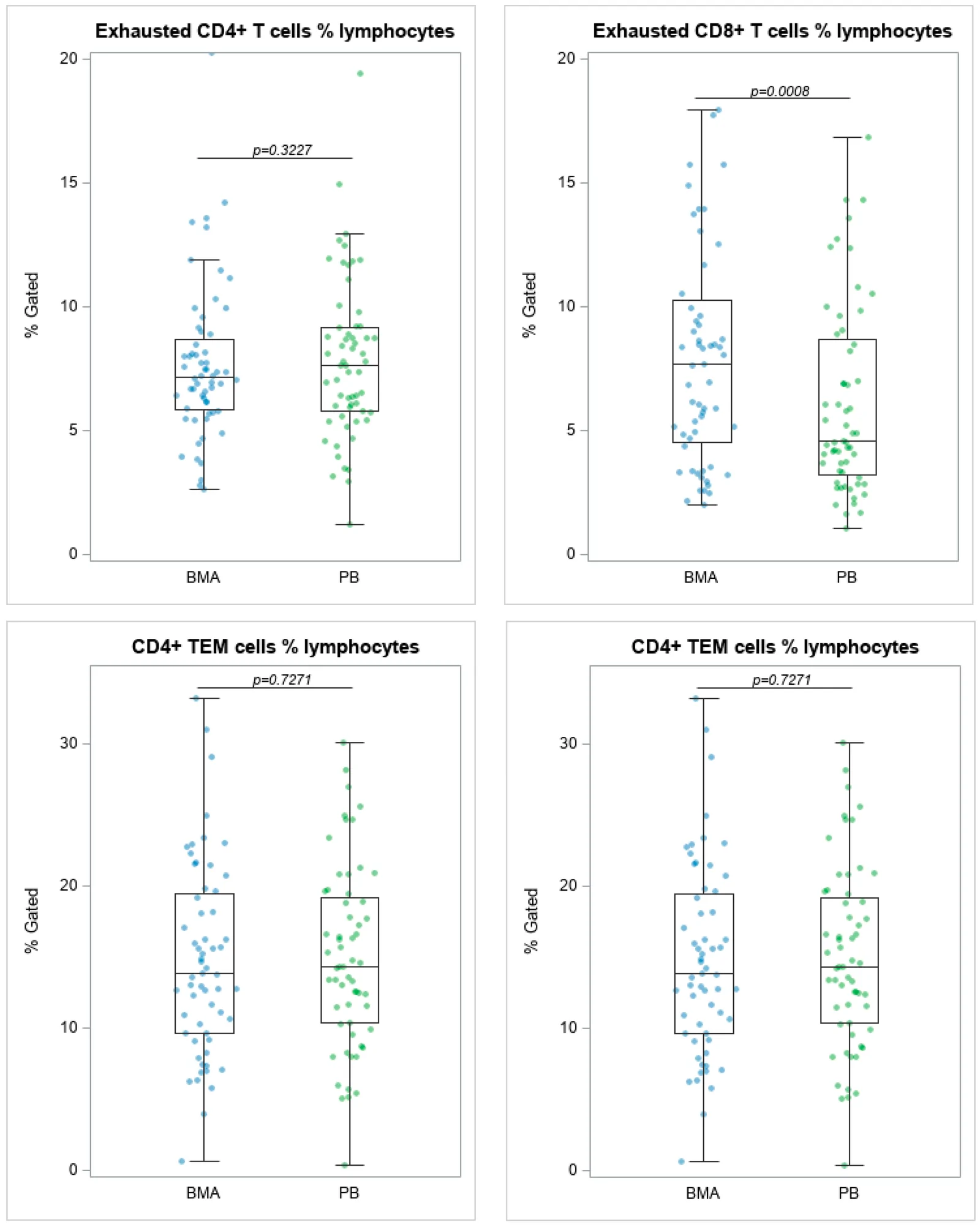
Exhausted and effector memory CD4^+^ and CD8^+^ T cell populations in the bone marrow aspirate (BMA) and paired peripheral blood (PB) of patients being evaluated for plasma cell disorders at Roswell Park Comprehensive Cancer Center, 2020–2024 (*n* = 60). While the PB and BMA have generally similar levels of exhausted and effector memory CD4^+^ T cells, the BMA has significantly higher levels of CD8^+^ exhausted and effector memory T cells than the PB. Error bars on whiskers represent 1.5 times the interquartile range.

Median PG marker values for the BMA and PB compartments are shown in Table 2 and for select markers in Figure 1.

Almost half of the markers examined have significantly different median values in the two compartments, showing that there is generally limited absolute agreement between the populations in the two compartments. This general lack of agreement in abundance is depicted in the PLS-DA score plot, which reveals a degree of separation between the two compartments (Figure S1). The largest absolute differences are in the CD8^+^ populations, particularly the effector memory and exhausted subsets, with more modest differences in CD4^+^, B cell, and other populations. The BMA favors CD8^+^ T cells, particularly activated and exhausted subsets—differences that are more marked in the symptomatic than asymptomatic patients. The PB favors CD4^+^ T cells, particularly naïve, TCM, and non-exhausted subsets. The abundance heat map of the eight most discriminatory markers reveals two major clusters among the PB and BMA samples, with the top cluster somewhat enriched for PB (34/59, 57.6%) and characterized by a higher relative abundance of total lymphocytes and CD4^+^ T cells and the bottom cluster somewhat enriched for BMA (34/57, 59.6%) and showing a higher relative abundance of CD8^+^ T and CD57^−^ NKT cells (Figure S2). Markers with the least absolute agreement between the PB and BMA, i.e., a statistically significant median difference of ≥10%, include γδ T cells (11%, *p* = 0.0050), CD8^+^ TEM (26%, *p* = 0.0008), CD8^+^ TEMRA (12%, *p* = 0.0068), exhausted CD8^+^ T cells (33%, *p* = 0.0008), CD57^−^ NKT cells (17%, *p* = 0.0019), CD8^+^ TEM/TCM (34%, *p* = 0.0008), CD8^+^ exhausted/non-exhausted (32%, *p* = 0.0008) (*higher in the BMA*); and total lymphocytes (16%, *p* = 0.0008), CD4^+^ TCM (10%, *p* = 0.0035), CD4/CD8 (14%, *p* = 0.0008) and Tregs/CD8^+^ T cells (10%, *p* = 0.0034) (*higher in the PB*).

Several markers have very good agreement between the PB and BMA, i.e., small (<5%) statistically non-significantly different medians in the BMA and PB. These include total T cells (2%, *p* = 0.0927), CD4^+^ TEM (2%, *p* = 0.7271), TEMRA (2%, *p* = 0.9418), Treg (1%, *p* = 0.8855), and exhausted (3%, *p* = 0.3227); CD8^+^ non-exhausted (1%, *p* = 0.5461); NCSM B cells (0%, *p* = 0.8855); NK cells (total [2%, *p* = 0.9884], CD57^+^ [1%, *p* = 0.3417], CD57^−^ [4%, *p* = 0.4690]); CD57^+^ NKT cells (3%, *p* = 0.3417); CD57^+^ LGLs (4%, *p* = 0.2212); and the following ratios: CD4^+^ naïve/memory (3%, *p* = 0.3227), CD4^+^ TEM/exhausted (3%, *p* = 0.9884), and B cells naïve/memory (1%, *p* = 0.9884).

### 3.3. BMA-PB Correlations

**Figure 2:**
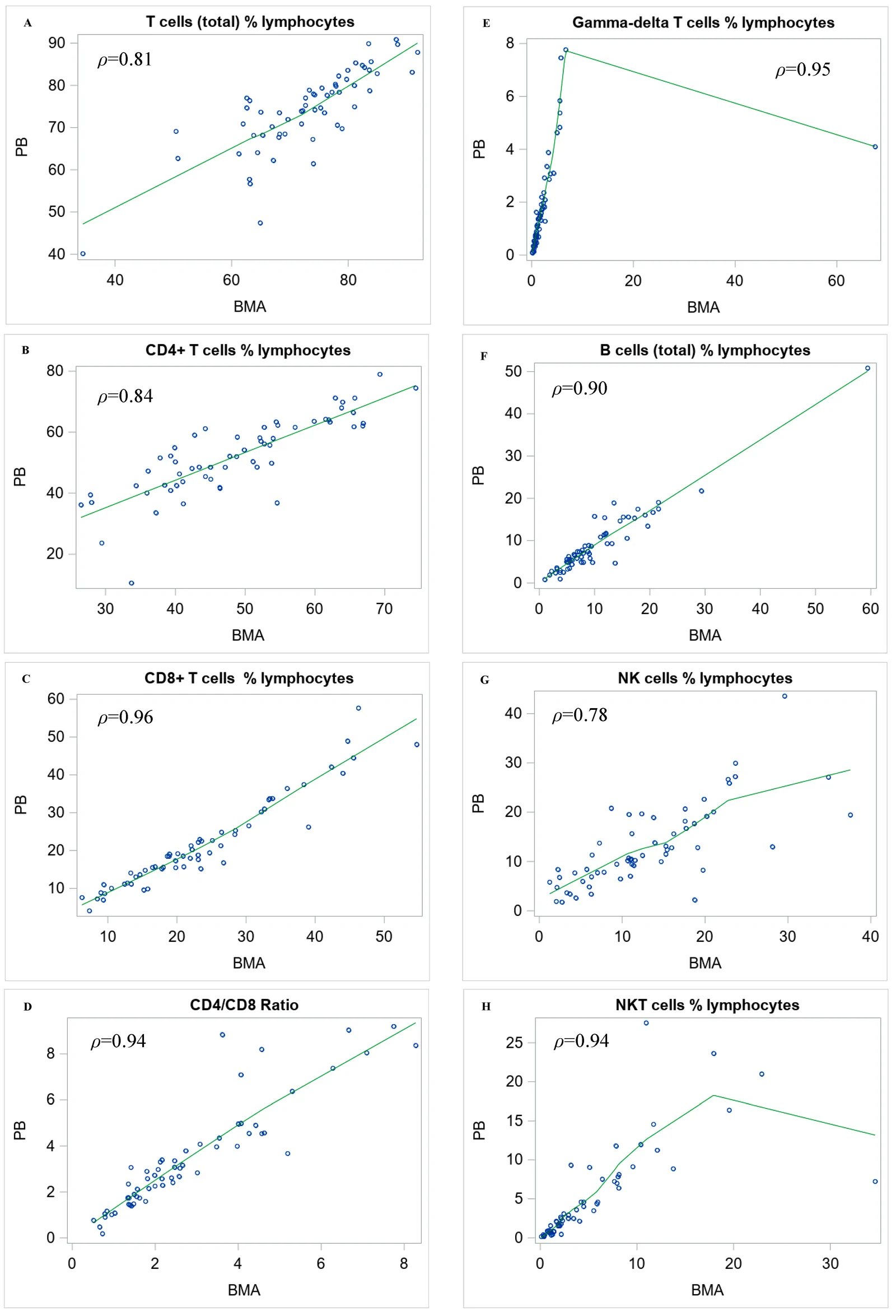
Scatter plots showing major lymphocyte populations as a percent of total lymphocytes in bone marrow and paired peripheral blood of patients being evaluated for plasma cell disorders at Roswell Park Comprehensive Cancer Center, 2020–2024 (*n* = 60). The strongest correlations between bone marrow and peripheral blood are in CD8^+^ T cells, gamma-delta T cells, and NKT cells. Locally weighed regression (LOESS) lines are in green. ρ = Spearman correlation coefficient. BMA = bone marrow aspirate. NKT = natural killer T. PB = peripheral blood.

The BMA and PB of all markers are significantly correlated, with all but two having very strong (ρ ≥ 0.90) or strong (0.70 ≤ ρ < 0.90) positive correlations, as shown in Table 2 and for select markers in Figure 2. Markers with the strongest correlations (ρ ≥ 0.95) include γδ T cells (ρ = 0.95), CD8^+^ T cells total (ρ = 0.96) and non-exhausted (ρ = 0.96), CD57^+^ NKTs (ρ = 0.97) and the CD4^+^ naïve/memory (ρ = 0.96) and B cells naïve/memory (ρ = 0.95) ratios. The two markers that are only moderately correlated in BMA and PB are CD4^+^ TEMRA (ρ = 0.66) and the ratio CD8^+^ TEM/TCM (ρ = 0.58).

### 3.4. Populations with Both Similar Abundance and High Correlation

The following populations have the most similar abundance (i.e., the closest absolute agreement) between the BMA and PB and are most strongly correlated: non-exhausted CD8^+^ T cells (1% higher in PB; ρ = 0.96), CD57^+^ NKTs (3% higher in PB; ρ = 0.97), the CD4^+^ naïve/memory ratio (3% higher in BMA, ρ = 0.96), and the B cells naïve/memory ratio (1% higher in PB, ρ = 0.95).

### 3.5. BMA-PB Correlations in Exploratory Subgroup Analyses

Most markers’ BMA-PB correlations do not differ significantly by subgroup, but there are some exceptions. Compared to non-obese patients (*n* = 30), obese patients (*n* = 30) have a significantly lower BMA-PB correlation in the CD8^+^ TEM/exhausted ratio (Δρ = 0.60, [0.25, 1.03]) (Table S3). Compared to patients whose BMA and PB samples were taken within one day of each other (*n* = 30), patients whose samples were taken more than one day apart (*n* = 30) have lower BMA-PB correlations in total T cells (Δρ = 0.24 [0.02, 0.56]), CD4^+^ naïve T cells (Δρ = 0.10 [0.00, 0.26]), Tregs (Δρ = 0.34 [0.10, 0.64]), CSM B cells (Δρ = 0.21 [0.02, 0.43]), total NK cells (Δρ = 0.28 [0.03, 0.62]), CD57^−^ NK cells (Δρ = 0.38 [0.11, 0.78]), and the CD4^+^ naïve/memory ratio (Δρ = 0.07 [0.01, 0.18]) (Table S4). Compared to asymptomatic patients, symptomatic patients have a lower BMA-PB correlation in exhausted CD8+ T cells (Δρ = 0.17 [0.01, 0.40]) (Table S5). Compared to older patients (*n* = 35), patients younger than 66 (*n* = 25) have a lower BMA-PB correlation in NCSM B cells (Δρ = 0.11 [0.02, 0.31]) (Table S6). Compared to patients whose BMA samples are more likely to be contaminated by blood (leukocyte count of ≤10,500 cells per μL), patients whose BMA samples are presumably less contaminated by blood (leukocyte count of >10,500 cells per μL) have a lower BMA-PB correlation in NK cells (Δρ = 0.32 [0.00, 0.76]) and CD57+ NK cells (Δρ = 0.24 [0.00, 0.60]) (Table S7). There are no significant differences in BMA-PB correlations by sex (Table S2).

### 3.6. Exploratory Analysis of Paired Lesion, BMA, and PB

As shown in Figure S3, in the one patient who had immunophenotyping performed on the PB, BMA, and a focal lesion, compared to the BMA, the PB is more reflective of the immune cell composition of the lesion for total T cells, CD8^+^ T cells, B cells, NK cells, and NKT cells. This is in spite of the fact that the PB draw was eight days before the BM and focal lesion biopsies. Only CD4^+^ T cells are similarly represented in the three compartments.

## 4. Discussion

The current analysis reveals that, within treatment-naïve individuals being evaluated for PCDs, the relative abundances of most lymphocyte populations, while showing generally limited absolute agreement between the two compartments, are highly correlated in the PB and BMA, independent of sex, obesity status, and age of the patient; whether the patient has symptomatic or asymptomatic disease; the amount of contamination of the BMA sample by blood (to partially but not fully eliminate variation from hemodilution due to unknown aspirate sampling order); and, to a lesser extent, the number of days in between the paired BMA-PB draws. The strength of the observed correlations suggests that lymphocyte populations in the PB compartment, though not definitive surrogates, might provide good candidates for predictive modeling of the analogous BMA populations. Moreover, a few key PB markers have both a similar absolute abundance (i.e., close agreement) and are highly correlated with their BMA counterparts, suggesting that, in this study, many individuals’ PB compartments are relatively similar to their BMA with regard to the abundance of non-exhausted CD8^+^ T cells, CD57^+^ NKTs, CD4^+^ naïve/memory ratio, and the B cells naïve/memory ratio. Although neither absolute agreement nor statistical correlation are sufficient evidence to say that the PB is a clinical surrogate for the BMA, especially given the fact that this analysis is based on samples which are unperturbed by treatment effects and does not include a longitudinal component, the results lay the groundwork for further examination of these two compartments in tandem.

### 4.1. Immune Changes in PCDs

The immune system is impacted in PCDs. Both the PB and BM of patients with MM contain higher levels of total T cells and NK cells than healthy individuals, and these changes are already apparent in the BM of individuals with MGUS. Moreover, in patients with SMM, the BM begins to show a loss of memory cytotoxic T cells and more markers of activation [24]. The BM of patients with newly diagnosed HRSMM had more naïve and memory CD4^+^ T cells, more NK cells, and more activated CD8^+^ T cells, but the cytotoxic T cells were also more dysfunctional. Importantly, the differences in immune cell composition between HRSMM patients’ and healthy individuals’ BM were largely similar to the differences observed comparing patients’ and healthy individuals’ PB [9]. Given the immune changes occurring all along the PCD spectrum, having the ability to understand what is happening in the BM through an examination of the PB is a potentially important clinical tool.

### 4.2. PB and BM Immune Composition Comparison

It is to be expected that the BM and PB would have correlated immune profiles, as there is movement of T cells between the PB and BM. For example, in mice, naïve T cells are primed while they traffic through the BM [25], and memory CD8^+^ cells migrate to the BM where they proliferate [26]. In fact, the BM is considered a reservoir for long-lived memory T cells [27]. Patients with acute myeloid leukemia (AML) have been shown to have strong BM-PB correlations for the CD4/CD8 ratio and effector T cell subsets [28].

In spite of the similarities caused by trafficking between the two environments, the PB and BM have important differences. In healthy adults, there is a lower level of T cells (% of total mononuclear cells) and a lower CD4/CD8 ratio but more expression of proliferative genes and more B cells in the BM than PB. Comparing healthy individuals and patients with precursor PCDs, the patients had lower PB levels of CD4^+^ T cells, Tregs, naïve CD8^+^ T cells, NKs, and B cells and elevated BM levels of Tregs, CD8^+^ T cells, and NKs [29], suggesting that the relationships between certain immune cell populations in the PB and BM are impacted in PCD precursors. Our study gives additional insight into what populations diverge the most between the BM and PB in patients with PCDs.

### 4.3. Comparison of Extant Work to Current Study

Most of the extant work examining the BM and PB compartments has focused on comparisons of typical abundance; the current study recapitulates some of these findings. Similar to healthy individuals, the patients in the current study have lower levels of T cells (though non-significantly) and a lower CD4/CD8 ratio and higher levels of B cells in their BMA than PB [30,31,32]. Furthermore, healthy adults have more expression of proliferative genes in the BM. Although we did not test for markers of proliferation, the BMA of our patients shows a more activated CD8^+^ profile and more NKT cells negative for CD57—the marker of replicative senescence.

The limited work showing BM-PB correlations, rather than absolute levels, has been conducted in healthy individuals and patients with HRSMM and AML, where, consistent with our findings, the PB and BM have been shown to be correlated in their CD4/CD8 ratio, effector cells, and certain other immune markers [9,28]. Though these findings are consistent with what was observed in the current study, we present data on more and different immune markers and consider factors that might impact the observed correlations.

### 4.4. Discussion of Key Findings

There are notable differences in how some of the T cell subpopulations differ in abundance (i.e., absolute agreement) by compartment. While levels of CD8^+^ T cells expressing no exhaustion markers are found in a similar abundance and are very strongly correlated in the PB and BMA, CD8^+^ T cells expressing two or more exhaustion markers are substantially more abundant in the BMA than PB—a difference that is somewhat more pronounced in the symptomatic than asymptomatic patients. This higher abundance of exhausted CD8^+^ T cells, along with significantly and substantially higher levels of CD8^+^ TEM cells and more modestly higher levels of CD8^+^ TEMRA cells, proliferating (CD57^−^) NKTs, and γδ T cells, suggests that the BM environment is more capable of rapid immune response than the PB but is also more highly antigen exposed—consistent with it being the site of disease in these patients. Conversely, the PB has a higher abundance (though the absolute differences are less marked than those found at higher abundance in the BMA) of CD4^+^ T cells, particularly naïve, TCM, and cells expressing no exhaustion markers, suggesting a less antigen exposed and exhausted immune milieu than the BMA, where most of the disease burden is located. Importantly, though the aforementioned immune cell subsets all have significantly higher abundance in the PB or BMA, they are all strongly or very strongly correlated in the two compartments, suggesting they should be explored as candidates for predictive modeling.

Two key populations have very similar abundance and are very strongly correlated in the BMA and PB: non-exhausted CD8^+^ T cells and CD57^+^ NKTs. Non-exhausted CD8^+^ T cells are critical to effective treatment with IMiDs and monoclonal antibodies, which work, in part, by activating CD8^+^ T cells. Chronic activation of T cells caused by bispecific antibodies can lead to T cell exhaustion, making a PB-based proxy for the non-exhausted reserve in the BM compartment a particularly relevant biomarker. Though they have limited proliferative capacity, CD57^+^ NKT cells are mature highly cytotoxic lymphocytes, making knowledge of their abundance in the MM TIME a useful component of our overall understanding of a patient’s cytotoxic capacity.

The few markers with a less than strong correlation between the two compartments are worth examining in closer detail. Two measures of effector function—the CD8^+^ TEM/TCM ratio and CD4^+^ TEMRA cells—are only moderately correlated in the two compartments in the combined sample, suggesting that, in patients with PCDs, the BM and PB are subject to compartment-specific factors influencing their effector profile. A prospective, serial analysis would be needed to explore whether the weaker BMA-PB correlations in these two measures of effector function are due to other factors.

Subgroup analyses, though limited by small sample sizes in each stratum, do not reveal any substantial stratum-specific differences in the strong paired BMA-PB correlations observed in the combined sample. This provides evidence that correlations between the immune cell populations in the BM and PB compartments might not be significantly impacted by sex, obesity, PCD stage, and age. Moreover, the statistically similar BMA-PB correlations in all but two markers between patients whose BMA samples were likely to be relatively uncontaminated by blood and patients with likely higher BMA contamination by blood—a limitation of the current study design, which has not controlled for order of the aspirate pull—suggests that the observed similarity in immune profiles of the paired BMA and PB samples is not primarily an artifact of contamination by blood that might occur during biopsy. The lower BMA-PB correlation in the CD8^+^ TEM/exhausted ratio in obese patients (vs. non-obese) and in exhausted CD8+ T cells in symptomatic (vs. asymptomatic) suggest that obesity and symptom burden could cause differential effects on cytotoxic T cells in the two compartments. As would be expected, a larger number of days between the paired samples seems to cause the two compartments’ immune profiles to become less comparable on some—but not most—measures.

In an illustrative single-patient exploratory analysis with paired data from all three compartments, the PB is generally more representative of the immune profile of the focal lesion than the BMA is, in spite of the BM and lesion biopsies being performed on the same day—eight days after the PB draw was done. Additional work is needed to expand the three-compartment comparison to more patients in order to facilitate statistical inference about the relative merit of the PB as a surrogate for focal lesions in MM.

In conclusion, the current study presents evidence that the pre-treatment PB has the potential to provide a window, either directly or through predictive modeling, into a broad array of immune markers in the individual patient’s pre-treatment BMA. Considering the prognostic importance of several of these BMA immune cell populations in PCDs [9,10,11,12,13,14,15,16], these findings could lead to more effective PB-based serial monitoring of immune health and treatment responses in PCDs; however, longitudinal analyses conducted in patients at various points along the treatment continuum and including more paired lesions are needed in order to understand the full capability of the PB as a surrogate for the TIME in PCDs, as well as to examine these findings in relation to the depth of response and progression-free survival.

## 5. Conclusions

The proper diagnosis and management of MM require use of BM biopsies at multiple timepoints. The current study provides evidence that features of the pre-treatment immune landscape in the BM can be interrogated by means of PB immunophenotyping, providing an accurate picture of the microenvironment relevant to this cancer. Given that abundances of several important immune cell populations and functional subsets are strongly correlated in the two compartments, these findings suggest that, as ancillary to the standard-of-care BM biopsies, there could be a role for PB immunophenotyping in addition to/in between the highly invasive biopsies. Further research is needed to understand what features of the PB immune profile might serve as harbingers of disease-related changes in the BM and to establish the prognostic relevance of these findings.

## Figures and Tables

**Table 1:** Descriptive characteristics of 60 patients being evaluated for plasma cell disorders at Roswell Park Comprehensive Cancer Center (2020–2024).

	Mean (SD) *n* (%)				*p*-Value ‡
Characteristics	All Subjects	MM	Precursor †	Other ∫	
	*n* = 60	*n* = 36	*n* = 16	*n* = 8	
Age at immune profiling (years)	66.5 (9.9)	66.3 (9.6)	69.4 (8.8)	61.9 (12.5)	0.2151
Body mass index (kg/m^2^)	29.8 (5.9)	29.4 (5.8)	29.8 (5.9)	31.8 (7.0)	0.5831
Days between PB, BMA profiling	3.8 (4.4)	3.2 (4.0)	5.4 (5.1)	3.6 (4.3)	0.2596
Sex Male Female	41 (68.3) 19 (31.7)	23 (63.9) 13 (36.1)	12 (75.0) 4 (25.0)	6 (75.0) 2 (25.0)	0.6631
Race/Ethnicity Non-Hispanic White Non-Hispanic Black Other race/ethnicity	45 (75.0) 10 (16.7) 5 (8.3)	30 (83.3) 2 (5.6) 4 (11.1)	10 (62.5) 6 (37.5) 0 (0.0)	5 (62.5) 2 (25.0) 1 (12.5)	0.0229
Smoking status Never Former Current	34 (56.7) 24 (40.0) 2 (3.3)	21 (58.3) 14 (38.9) 1 (2.8)	7 (43.8) 8 (50.0) 1 (6.3)	6 (75.0) 2 (25.0) 0 (0.0)	0.6088
Bone marrow plasma cell %	29.1 (26.2)	41.2 (23.7)	10.5 (12.0)	11.9 (27.6)	<0.0001
Serum M protein (g/dL)	1.9 (2.2)	2.4 (2.4)	0.6 (0.9)	2.4 (3.0)	0.0206
Sub-type IgG IgA IgM Biclonal Light-chain only	27 (51.9) 7 (13.5) 3 (5.8) 3 (5.8) 12 (23.1)	19 (52.8) 5 (13.9) 0 (0.0) 1 (2.8) 11 (30.6)	8 (50.0) 2 (12.5) 3 (18.8) 2 (12.5) 1 (6.3)	N/A	0.0005
Stage (R-ISS) I II III Unstaged	N/A	13 (26.1) 18 (50.0) 4 (11.1) 1 (2.8)	N/A	N/A	N/A
High-risk cytogenetics (>0 marker)	N/A	8 (22.9)	N/A	N/A	N/A
Serum calcium >2.75 mmol/L	N/A	4 (11.1)	N/A	N/A	N/A
Serum creatinine >2.0 mg/dL	N/A	8 (22.2)	N/A	N/A	N/A
Hemoglobin <10.0 g/dL	N/A	10 (27.8)	N/A	N/A	N/A
Lytic bone lesions	N/A	26 (72.2)	N/A	N/A	N/A
No CRAB features	N/A	6 (16.7)	N/A	N/A	N/A

‡ *p*-values from Student’s *t* tests of differences in mean, chi-square tests of independence, or Fisher’s exact test, as appropriate; † Precursor includes MGUS, SMM. ∫ Other includes negative for MM (*n* = 2), AL amyloidosis with unknown underlying condition (*n* = 2), AL amyloidosis with underlying MGUS (*n* = 1), plasma cell leukemia (*n* = 1), plasmacytoma (*n* = 1), not definitive (*n* = 1). Abbreviations: AL = light chain amyloidosis; BMA = bone marrow aspirate; dL = deciliter; L = liter; M = monoclonal; MGUS = monoclonal gammopathy of undetermined significance; MM = multiple myeloma; mmol = millimole; *n* = number; PB = peripheral blood; R-ISS = Revised International Staging System; SD = standard deviation; SMM = smoldering multiple myeloma.

**Table 2:** Comparison of immune profiles of paired bone marrow aspirate and peripheral blood of 60 patients being evaluated for plasma cell disorders at Roswell Park Comprehensive Cancer Center (2020–2024).

	Bone Marrow Aspirate	Peripheral Blood	Bone Marrow Aspirate vs. Peripheral Blood		Spearman Correlations		
	Median (IQR)	Median (IQR)	BMA-PB (%)	*p*-Value † ↕	ρ (95% CI)	Strength	*p*-Value ↕
Percent Gated #							
Lymphocytes, total	20.0 (15.6)	26.5 (15.3)	−4.1 (−16%)	0.0008 *	0.80 (0.69–0.88)	Strong	<0.0041 *
T cells, total	74.0 (13.7)	74.8 (11.3)	−1.8 (−2%)	0.0927	0.81 (0.71–0.88)	Strong	<0.0041 *
γδ^+^ T cells	1.4 (1.8)	1.3 (1.5)	0.1 (11%)	0.0050 *	0.95 (0.92–0.97)	Very Strong	<0.0041 *
CD4^+^ T cells							
Total	48.3 (15.9)	52.1 (18.8)	−4.1 (−8%)	0.0008 *	0.84 (0.74–0.90)	Strong	<0.0041 *
Naïve	19.1 (17.5)	20.5 (17.2)	−1.1 (−5%)	0.0310 *	0.94 (0.89–0.96)	Very Strong	<0.0041 *
TCM	10.7 (8.8)	12.4 (9.0)	−1.3 (−10%)	0.0035 *	0.81 (0.71–0.89)	Strong	<0.0041 *
TEM	13.8 (9.8)	14.3 (8.8)	0.3 (2%)	0.7271	0.83 (0.73–0.89)	Strong	<0.0041 *
TEMRA	1.6 (1.6)	1.4 (1.6)	0.0 (−2%)	0.9418	0.66 (0.49–0.79)	Moderate	<0.0041 *
Treg	4.3 (1.4)	4.3 (1.7)	−0.0 (−1%)	0.8855	0.74 (0.59–0.83)	Strong	<0.0041 *
Non-exhausted ↑	27.8 (15.9)	29.8 (17.2)	−2.8 (−9%)	0.0008 *	0.90 (0.83–0.94)	Strong	<0.0041 *
Exhausted ↓	7.2 (2.8)	7.6 (3.4)	−0.2 (−3%)	0.3227	0.81 (0.70–0.88)	Strong	<0.0041 *
CD8^+^ T cells							
Total	22.0 (14.1)	18.6 (11.9)	1.3 (7%)	0.0008 *	0.96 (0.93–0.97)	Very Strong	<0.0041 *
Naïve	3.5 (3.2)	2.8 (3.3)	0.2 (7%)	0.3227	0.87 (0.79–0.92)	Strong	<0.0041 *
TCM	1.3 (1.5)	1.6 (1.5)	−0.2 (−11%)	0.0826	0.71 (0.56–0.82)	Strong	<0.0041 *
TEM	6.1 (6.7)	4.5 (4.9)	1.2 (26%)	0.0008 *	0.79 (0.67–0.87)	Strong	<0.0041 *
TEMRA	6.9 (9.6)	5.9 (8.9)	0.7 (12%)	0.0068 *	0.94 (0.90–0.96)	Very Strong	<0.0041 *
Non-exhausted ↑	5.4 (5.6)	5.2 (6.1)	−0.0 (−1%)	0.5461	0.96 (0.93–0.98)	Very Strong	<0.0041 *
Exhausted ↓	7.7 (5.7)	4.6 (5.5)	1.5 (33%)	0.0008 *	0.86 (0.78–0.92)	Strong	<0.0041 *
B cells							
Total	8.1 (7.9)	7.0 (7.7)	0.5 (7%)	0.0008 *	0.90 (0.84–0.94)	Very Strong	<0.0041 *
Naïve	4.4 (7.5)	4.0 (7.0)	0.2 (5%)	0.0310 *	0.94 (0.91–0.97)	Very Strong	<0.0041 *
Memory	2.2 (1.6)	1.9 (2.1)	0.1 (7%)	0.0656	0.82 (0.71–0.89)	Strong	<0.0041 *
NCSM	0.7 (0.9)	0.7 (1.0)	0.0 (0%)	0.8855	0.93 (0.89–0.96)	Very Strong	<0.0041 *
CSM	1.3 (1.1)	1.1 (1.1)	0.1 (5%)	0.0367 *	0.81 (0.69–0.88)	Strong	<0.0041 *
NK/NKT cells							
NK, total	11.3 (11.9)	11.0 (11.2)	0.2 (2%)	0.9884	0.78 (0.65–0.86)	Strong	<0.0041 *
NK, CD57^+^	6.1 (6.4)	5.9 (7.6)	−0.1 (−1%)	0.3417	0.79 (0.67–0.87)	Strong	<0.0041 *
NK, CD57^−^	4.8 (4.6)	4.7 (4.5)	0.2 (4%)	0.4690	0.76 (0.63–0.85)	Strong	<0.0041 *
NKT, total	2.6 (6.5)	2.5 (6.5)	0.2 (7%)	0.1243	0.94 (0.90–0.96)	Very Strong	<0.0041 *
NKT, CD57^+^	1.1 (4.9)	1.3 (5.3)	−0.0 (−3%)	0.3417	0.97 (0.95–0.98)	Very Strong	<0.0041 *
NKT, CD57^−^	1.4 (1.6)	1.0 (1.4)	0.2 (17%)	0.0019 *	0.84 (0.74–0.90)	Strong	<0.0041 *
LGL, CD57^+^	4.7 (9.1)	4.0 (8.4)	0.2 (4%)	0.2212	0.94 (0.91–0.97)	Very Strong	<0.0041 *
Ratios of Absolute Cell Counts							
CD4/CD8	2.17 (2.54)	2.86 (2.70)	−0.41 (−14%)	0.0008 *	0.94 (0.90–0.96)	Very Strong	<0.0041 *
CD4^+^ Tregs/CD8^+^ T cells	0.21 (0.18)	0.24 (0.22)	−0.02 (−10%)	0.0034 *	0.89 (0.83–0.94)	Strong	<0.0041 *
CD4^+^ Naïve/memory ∫	0.78 (1.03)	0.74 (0.86)	0.02 (3%)	0.3227	0.96 (0.93–0.97)	Very Strong	<0.0041 *
CD4^+^ TEM/TCM	1.25 (0.9)	1.02 (0.97)	0.11 (10%)	0.0726	0.75 (0.61–0.84)	Strong	<0.0041 *
CD4^+^ Exhausted/non-exhausted ↓↑	0.25 (0.2)	0.23 (0.19)	0.01 (6%)	0.0826	0.90 (0.84–0.94)	Very Strong	<0.0041 *
CD4^+^ TEM/Exhausted ↓	1.84 (0.59)	1.84 (0.90)	0.06 (3%)	0.9884	0.75 (0.62–0.85)	Strong	<0.0041 *
CD8^+^ Naïve/memory ∫	0.42 (0.63)	0.47 (0.86)	−0.04 (−8%)	0.0970	0.84 (0.75–0.90)	Strong	<0.0041 *
CD8^+^ TEM/TCM	4.92 (5.61)	3.14 (4.91)	1.07 (34%)	0.0008 *	0.58 (0.38–0.73)	Moderate	<0.0041 *
CD8^+^ Exhausted/non-exhausted ↓↑	1.38 (1.85)	1.02 (1.42)	0.32 (32%)	0.0008 *	0.90 (0.84–0.94)	Very Strong	<0.0041 *
CD8^+^ TEM/Exhausted ↓	0.85 (0.6)	0.86 (0.68)	−0.04 (−5%)	0.2771	0.71 (0.55–0.82)	Strong	<0.0041 *
B cells Naïve/memory	1.94 (3.51)	2.01 (3.34)	−0.03 (−1%)	0.9884	0.95 (0.91–0.97)	Very Strong	<0.0041 *

Symbols: # Lymphocytes stated as a percent of total leukocytes; all other populations stated as a percent of total lymphocytes. † Median differences tested by Wilcoxon signed-rank test. ↕ *p*-values corrected for false discovery rate using the Benjamini–Hochberg procedure. * *p* ≤ 0.05 after correction for false discovery rate. ↑ Non-exhausted: not expressing any of the following exhaustion markers: PD1, TIGIT, LAG3, TIM3. ↓ Exhausted: Expressing two or more of the following exhaustion markers: PD1, TIGIT, LAG3, TIM3. ∫ Memory = TEM + TCM. Abbreviations: BMA = bone marrow aspirate; CD = cluster of differentiation; CI = confidence interval; CSM = class-switched memory; γδ = gamma-delta T cells; IQR = interquartile range; *n* = number; LGL = large granular lymphocytes; NCSM = non-class-switched memory; NK = natural killer cells; NKT = natural killer T cells; PB = peripheral blood; ρ = Spearman correlation coefficient; TCM = central memory T cells; TEM = effector memory T cells; TEMRA = terminally differentiated TEM (TEM re-expressing CD45RA).

## Data Availability

The data that support the findings of this study are not publicly available due to restrictions set by the study protocol. Requests for access to the data may be directed to the corresponding author.

## Supplementary Materials

The following supporting information can be downloaded at https://www.mdpi.com/article/10.3390/cancers18213428/s1, Table S1: Cell populations and immunophenotyping markers; File S1: Flow cytometric gating strategy and data analysis for flow panel employed in paired PB-BMA analysis; File S2: Flow panel reagent list; Table S2: Comparison of immune profiles of paired bone marrow aspirate and peripheral blood of 60 patients, stratified by sex, being evaluated for plasma cell disorders at Roswell Park Comprehensive Cancer Center (2020–2024); Table S3: Comparison of immune profiles of paired bone marrow aspirate and peripheral blood of 60 patients, stratified by obesity status, being evaluated for plasma cell disorders at Roswell Park Comprehensive Cancer Center (2020–2024); Table S4: Comparison of immune profiles of paired bone marrow aspirate and peripheral blood of 60 patients, stratified by time between blood draw and bone marrow biopsy, being evaluated for plasma cell disorders at Roswell Park Comprehensive Cancer Center (2020–2024); Table S5: Comparison of immune profiles of paired bone marrow aspirate and peripheral blood of 60 patients, symptomatic vs. asymptomatic, being evaluated for plasma cell disorders at Roswell Park Comprehensive Cancer Center (2020–2024); Table S6: Comparison of immune profiles of paired bone marrow aspirate and peripheral blood of 60 patients, stratified by age, being evaluated for plasma cell disorders at Roswell Park Comprehensive Cancer Center (2020–2024); Table S7. Comparison of immune profiles of paired bone marrow aspirate and peripheral blood of 60 patients, stratified by bone marrow cellularity, being evaluated for plasma cell disorders at Roswell Park Comprehensive Cancer Center (2020–2024); Figure S1. Partial least squares discriminant analysis (PLS-DA) score plot of the two most explanatory components; Figure S2. Heatmap of the top 25% of markers contributing to the first PLS-DA component; Figure S3. Paired PB-lesion and BMA-lesion comparison in one patient.
