# Supplementary material for: Immunophenotyping of Paired Bone Marrow and Peripheral Blood: Implications for Monitoring Plasma Cell Disorders
Source: Cancers (Basel). 2026 Oct 23;18(21):3428. doi: 10.3390/cancers18213428 (PMC13761744; doi:10.3390/cancers18213428)
Supplement: Supplementary file 1 [file cancers-18-03428-s001.zip › File S1 - Gating Strategy for Flow Panel 10.2.25.pdf]

File S1: Flow Cytometric Gating Strategy and Data Analysis for Flow Panel Employed in Paired PB-BM Analysis

In collective, five 8-color immunophenotyping panels are evaluated, whose purposes and compositions are detailed in **Table 1**, below. These panels allow for the discrimination and quantification of major lymphocyte distributions; and also permit characterization of B cell and T cell antigen experience profiles.

Each 'tube' of the assay serves slightly different functions; however, there are several areas of consistency in the analysis approach for each tube. These shall be sequentially detailed, with redundant components of analytical overlap noted.

The primary objective for **ADA** is to obtain a flow cytometric measure of sample viability; for the sake of brevity this analysis has not been described. Instead, the analyses germane to the quantification lymphocyte subsets **(V195**, **V196**, **V197**, and **V198)** shall be described in detail.

Table 1: **Immunophenotyping Panel Composition**

Tube Name: ADA
Purpose: Establish Autofluorescence Intensity Thresholds for each Detector; Measure Sample Viability

Fluorochrome:	BB515	PE	PerCPCy5.5	PECy7	APC	APCH7	BV421	BV510
Antigen:						CD45		LDA

Tube Name: V195
Purpose: Measure T Cell Subset Frequencies; Establish FMO Intensity Thresholds for each Detector used in Measuring T Cell Exhaustion Markers

Fluorochrome:	BB515	PE	PerCPCy5.5	PECy7	APC	APCH7	BV421	BV510
Antigen:	CD8			CD3		CD45		CD4

Tube Name: V196
Purpose: Measure T Cell Subset Frequencies; Measure Frequencies of T Cell Exhaustion Markers

Fluorochrome:	BB515	PE	PerCPCy5.5	PECy7	APC	APCH7	BV421	BV510
Antigen:	CD8	CD336 (TIM-3)	LAG-3	CD3	TIGIT	CD45	CD279 (PD-1)	CD4

Tube Name: V197
Purpose: Measure T Cell Subset Frequencies; Treg Frequencies; Characterize T Cell Antigen Experience Profile

Fluorochrome:	BB515	PE	PerCPCy5.5	PECy7	APC	APCH7	BV421	BV510
Antigen:	CD25	CD197 (CCR7)	CD8	CD3	CD45RA	CD45	CD127	CD4

Tube Name: V198
Purpose: Measure B Cell and NK Cell Subset Frequencies; Characterize B Cell Antigen Experience Profile

Fluorochrome:	FITC	PE	PerCPCy5.5	PECy7	APC	APCH7	BV421	BV510
Antigen:	CD57<br>IgD	CD16<br>CD56	TCR γδ	CD3	CD27	CD45	CD33	CD19

V195 and V196 Analysis

The first four plots represented in **Figure A** are intended to restrict the data analysis to 'clean' lymphocytes by eliminating as much irrelevant data as possible from the analysis. To accomplish the initial data clean-up, all collected events are displayed using a bivariate plot of FTIM(1024) *vs.* FSC-A; where rectangular region **(R1)** is used to circumscribe all events in fluidic continuity, as a function of normalized chronology. Events that satisfy this selection event are gated to a bivariate plot of FSC-H *vs.* FSC-A, where trapezoidal region **(R2)** is used to circumscribe singlet events; thus removing non-singlet (*i.e.* doublet, triplet, *etc.*) events from the analysis.

Events meeting the gate definition of **(R1&R2)** are gated to a bivariate plot of Ex SSC-A *vs.* CD45 AH7 to reveal the CD45 expression profiles for interrogated leukocytes in the context of their orthogonally-scattered light characteristic; thus elucidating several discrete cellular distributions. Therein, rectangular region **(R3)** is used to circumscribe all CD45+ events, and

lymphocyte events **(R4)** are separately resolved and circumscribed.

Thereafter, events meeting the Boolean gate definition of **(R1&R2&R3&R4)** are gated to a bivariate plot of FSC-A *vs.* Ex SSC-A, where events are displayed as a function of their light scatter characteristics. Elliptical region **(R5)** is used to circumscribe classically recognized lymphocyte events by virtue of their distribution in light scatter-space.

The frequency of events satisfying the gate definition of **(R1&R2&R3&R4&R5)** are classified as 'lymphocytes', and this gate serves as the denominator for subsequent calculations used to quantify the frequency of cellular populations (*i.e.* the percentage of total lymphocytes), unless otherwise noted.

The first objective of the analysis is to classify T Helper (CD3+/CD4+) and Cytotoxic (CD3+/CD8+) T cells, and separately to measure the percentage of these T cell subsets which express any combination of the checkpoint molecules: TIM-3, LAG-3, TIGIT, and PD-1. To accomplish this, 'lymphocytes' **(R1&R2&R3&R4&R5)** are gated to a bivariate plot of Ex SSC-A *vs.* CD3 PECy7, where rectangular region **(R6)** is used to circumscribe T-lymphocyte events by virtue of their expression of CD3. Thereafter, events satisfying the gate definition of **(R1&R2&R3&R4&R5&R6)** are gated to the following bivariate plots:

- CD8 BB515 *vs.* CD4 BV510: where quadstat regions **(R7)**, **(R8)**, **(R9)**, and **(R10)** are used to quantify the frequencies of CD3+ T cells expressing any combination of CD8 or CD4.
- Ex SSC-A *vs.* PE-A (or TIM-3 PE): where rectangular region **(R11)** is used to circumscribe events that are defined as 'positive' in the PE detector. The location of **(R11)** in Cartesian space is set using the **V195** control tube, which discriminates TIM-3 negative from TIM-3 positive events in the **V196** tube.

- Ex SSC-A *vs.* PerCPCy5.5-A (or LAG-3 PerCPCy5.5): where rectangular region **(R12)** is used to circumscribe events that are defined as 'positive' in the PerCPCy5.5 detector. The location of **(R12)** in Cartesian space is set using the **V195** control tube, which discriminates LAG-3 negative from LAG-3 positive events in the **V196** tube.

- Ex SSC-A *vs.* APC-A (or TIGIT APC): where rectangular region **(R13)** is used to circumscribe events that are defined as 'positive' in the APC detector. The location of **(R13)** in Cartesian space is set using the **V195** control tube, which discriminates TIGIT negative from TIGIT positive events in the **V196** tube.

- Ex SSC-A *vs.* BV421-A (or PD-1 BV421): where rectangular region **(R14)** is used to circumscribe events that are defined as 'positive' in the APC detector. The location of **(R14)** in Cartesian space is set using the **V195** control tube, which discriminates PD-1 negative from PD-1 positive events in the **V196** tube.

Figure A: Selected Data Plots Employed for the V195 and V196 Analyses

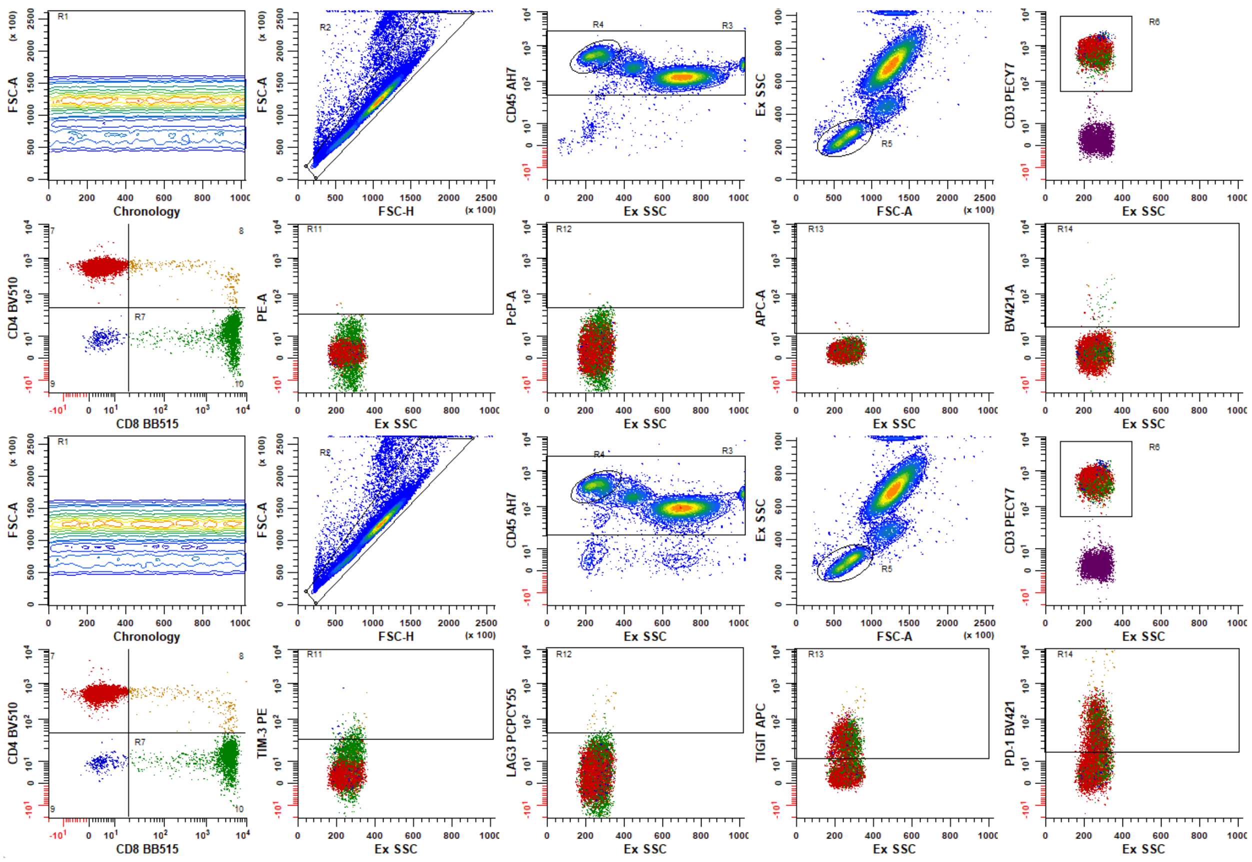

V197 Analysis:

As with **V195** and **V196**; the **V197** evaluation employs the same initial gating structure for the refinement of data; and for the classification of lymphocytes **(R1&R2&R3&R4&R5)** and CD3+ T cells **(R1&R2&R3&R4&R5&R6).** Thereafter, events satisfying the gate definition of **(R1&R2&R3&R4&R5&R6)** are gated to the following bivariate plots, illustrated in **Figure B**:

- CD8 BB515 *vs.* CD4 BV510: where quadstat regions **(R7)**, **(R8)**, **(R9)**, and **(R10)** are used to quantify the frequencies of CD3+ T cells expressing any combination of CD8 or CD4.

- CD4+ T cells **(R1&R2&R3&R4&R5&R6&R7)** are subsequently gated to a bivariate plot of CD25 BB515 *vs.* CD127 BV421, where quadrilateral region **(R11)** is used to circumscribe CD25(br)/CD127(d) Treg events. Separately, CD4+ T cells are gated to a bivariate plot of CD45APC *vs.* CCR7 PE, where quadstat regions **(R24)**, **(R25)**, **(R26)**, and **(R27)** are used to quantify the frequencies of CD4+ T cells expressing any combination of CD45RA or CCR7.

- CD8+ T cells **(R1&R2&R3&R4&R5&R6&R10)** are gated to a bivariate plot of CD45APC *vs.* CCR7 PE, where quadstat regions **(R28)**, **(R29)**, **(R30)**, and **(R31)** are used to quantify the frequencies of CD8+ T cells expressing any combination of CD45RA or CCR7.

V198 Analysis:

As with **V195** and **V196**; the **V198** evaluation employs the same initial gating structure for the refinement of data; and for the classification of lymphocytes **(R1&R2&R3&R4&R5).** Thereafter, the events satisfying the gate definition of **(R1&R2&R3&R4&R5)** are gated to a bivariate plot of CD33 BV421 *vs.* CD19 BV510, where quadstat regions **(R6)**, **(R7)**, **(R8)**, and **(R9)** are used to quantify the frequency of lymphocytes expressing any combination of CD19 or CD33. Subsequently, as illustrated in **Figure B**:

- CD19+ B cells **(R1&R2&R3&R4&R5&R6)** are gated to a bivariate plot of CD57/IgD FITC *vs.* CD27 APC, where quadstat regions **(R10)**, **(R11)**, **(R12)**, and **(R13)** are used to quantify the frequencies of CD19+ B cells expressing any combination of IgD or CD27.

- Events that are negative for CD19 and CD33 **(R1&R2&R3&R4&R5&R8)** are gated to a bivariate plot of Ex SSC-A vs. CD3 PECy7, where rectangular region **(R14)** is used to circumscribe T-lymphocyte events by virtue of their expression of CD3. Thereafter, CD3 negative events **(R1&R2&R3&R4&R5&R8&!R14)** are gated to a bivariate plot of CD57/IgD FITC vs. CD16/CD56 PE, where quadstat regions **(R24)**, **(R25)**, **(R26)**, and **(R27)** are used to quantify the frequencies of CD3 negative lymphocytes expressing any combination of IgD or CD27. Separately, CD3+ events **(R1&R2&R3&R4&R5&R8&R14)** are gated to a bivariate plot of CD57/IgD FITC *vs.* CD16/CD56 PE, where quadstat regions **(R28)**, **(R29)**, **(R30)**, and **(R31)** are used to quantify the frequencies of CD3+ lymphocytes expressing any combination of IgD or CD27. Finally, CD3+ events **(R1&R2&R3&R4&R5&R8&R14)** are separately gated to a bivariate plot of CD27 APC *vs.* TCR δγ, where quadstat regions **(R15)**, **(R16)**, **(R17)**, and **(R18)** are used to quantify the frequencies of CD3+ lymphocytes expressing any combination of CD27 or TCR δγ.

Figure B: Selected Data Plots Employed for the V197 and V198 Analyses

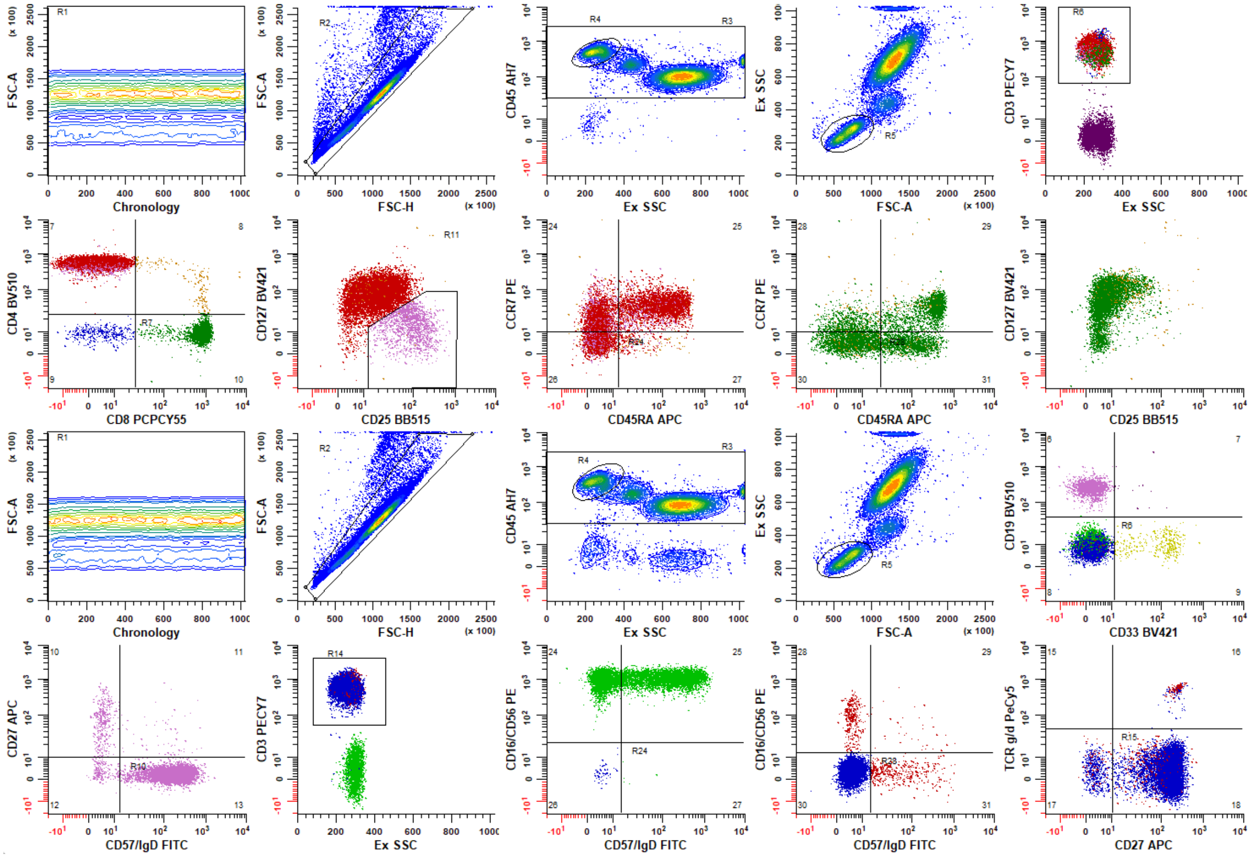
FSC-A
Chronology
FSC-H
CD45 AH7
Ex SSC
CD3 PECY7
CD4 BV510
CD8 PCPCY55
CD127 BV421
CD25 BB515
CCR7 PE
CD45RA APC
CD19 BV510
CD33 BV421
CD27 APC
CD57/IgD FITC
CD16/CD56 PE
TCR g/d PeCy5
