# Supplementary material for: Immunophenotyping of Paired Bone Marrow and Peripheral Blood: Implications for Monitoring Plasma Cell Disorders
Source: Cancers (Basel). 2026 Oct 23;18(21):3428. doi: 10.3390/cancers18213428 (PMC13761744; doi:10.3390/cancers18213428)
Supplement: Supplementary file 1 [file cancers-18-03428-s001.zip › File S2 - Flow Panel Reagent List 7.1.24.pdf]

File S2: Flow Panel Reagent List

Measurement	Fluorochrome	Vendor	Cat#	Clone
CD8	BB515	BD	564526	RPA-T8
CD366 TIM-3	PE	BioLegend	345006	E38-2E2
CD223 (LAG-3)	PcPCy5.5	BioLegend	369312	11C3C65
CD3	PECy7	Beckman Coulter	6607100	UCHT1
TIGIT	APC	Ebioscience	17-9500-42	MBSA43
CD45	APCH7	BD	641408	2D1
CD279 (PD-1)	BV421	BioLegend	329920	EH12-2H7
CD4	BV510	BD	562970	SK3
CD25	BB515	BD	564467	2A3
CD197 (CCR7)	PE	BD	560765	150503
CD8	PcPCy5.5	BD	341049	SK1
CD45RA	APC	BD	550855	HI100
CD127	BV421	BD	562436	HIL-7R-M21
CD57	FITC	Beckman Coulter	IM0466	NC1
IgD	FITC	BD Pharmingen	555778	IA6-2
CD16	PE	BD	340705	B73.1
CD56 - RD1	PE	Beckman Coulter	6603067	NKH-1
TCR gd	PECy5	Beckman Coulter	IM2662U	IMMU510
CD27	APC	BD	337169	L128
CD33	BV421	BioLegend	303416	WM53
CD19	BV510	BioLegend	302242	HIB19
