# Supplementary material for: Immunophenotyping of Paired Bone Marrow and Peripheral Blood: Implications for Monitoring Plasma Cell Disorders
Source: Cancers (Basel). 2026 Oct 23;18(21):3428. doi: 10.3390/cancers18213428 (PMC13761744; doi:10.3390/cancers18213428)
Supplement: Supplementary file 1 [file cancers-18-03428-s001.zip › Table S1. Immunophenotyping summary.pdf]

Table S1. Cell populations and immunophenotyping markers

The panel employed in this analysis included reagents to detect the following markers: CD45, CD3, CD4, CD8, TCR gamma/delta, CD25, CD45RA, CCR7, CD127, TIM-3, LAG-3, TIGIT, PD-1, CD19, CD27, IgD, CD16, CD56, CD57, CD33. A lymphocyte gate based on CD45 staining and light scatter was used to define the percent gated values. The following populations were immunophenotyped using the stated markers. Populations with a ✓mark in the last column were included in the current analysis. Exhausted populations with an * are included as part of the total exhausted.

**Population**	**Markers**	**Included in Current Analysis**
**Lymphocytes, total**	CD45^{+} plus light scatter	✓
**T cells**		
Total	CD45^{+} CD3^{+}	✓
Gamma/delta ($\gamma\delta$)$^{+}$	CD45^{+} CD3^{+} TCR gamma/delta^{+}	✓
**CD4^{+} T cells**		
Total	CD45^{+} CD3^{+} CD4^{+}	✓
Naïve	CD45^{+} CD3^{+} CD4^{+} CD45RA^{+} CCR7^{+}	✓
Central memory (TCM)	CD45^{+} CD3^{+} CD4^{+} CD45RA^{-} CCR7^{+}	✓
Effector memory (TEM)	CD45^{+} CD3^{+} CD4^{+} CD45RA^{-} CCR7^{-}	✓
Terminally differentiated effector memory (TEMRA)	CD45^{+} CD3^{+} CD4^{+} CD45RA^{+} CCR7^{-}	✓
Regulatory (Treg)	CD45^{+} CD3^{+} CD4^{+} CD25^{+} CD127(dim)	✓
**CD4^{+} T cells expressing exhaustion markers**		
Non-exhausted	CD45^{+} CD3^{+} CD4^{+} TIM3^{-} LAG3^{-} TIGIT^{-} PD1^{-}	✓
TIM3 only	CD45^{+} CD3^{+} CD4^{+} TIM3^{+} LAG3^{-} TIGIT^{-} PD1^{-}	
LAG3 only	CD45^{+} CD3^{+} CD4^{+} TIM3^{-} LAG3^{+} TIGIT^{-} PD1^{-}	
TIGIT only	CD45^{+} CD3^{+} CD4^{+} TIM3^{-} LAG3^{-} TIGIT^{+} PD1^{-}	
PD1 only	CD45^{+} CD3^{+} CD4^{+} TIM3^{-} LAG3^{-} TIGIT^{-} PD1^{+}	
TIM3/LAG3	CD45^{+} CD3^{+} CD4^{+} TIM3^{+} LAG3^{+} TIGIT^{-} PD1^{-}	*
TIM3/TIGIT	CD45^{+} CD3^{+} CD4^{+} TIM3^{+} LAG3^{-} TIGIT^{+} PD1^{-}	*
TIM3/PD1	CD45^{+} CD3^{+} CD4^{+} TIM3^{+} LAG3^{-} TIGIT^{-} PD1^{+}	*
LAG3/TIGIT	CD45^{+} CD3^{+} CD4^{+} TIM3^{-} LAG3^{+} TIGIT^{+} PD1^{-}	*
LAG3/PD1	CD45^{+} CD3^{+} CD4^{+} TIM3^{-} LAG3^{+} TIGIT^{-} PD1^{+}	*
TIGIT/PD1	CD45^{+} CD3^{+} CD4^{+} TIM3^{-} LAG3^{-} TIGIT^{+} PD1^{+}	*
TIM3/LAG3/TIGIT	CD45^{+} CD3^{+} CD4^{+} TIM3^{+} LAG3^{+} TIGIT^{+} PD1^{-}	*
TIM3/LAG3/PD1	CD45^{+} CD3^{+} CD4^{+} TIM3^{+} LAG3^{+} TIGIT^{-} PD1^{+}	*
TIM3/TIGIT/PD1	CD45^{+} CD3^{+} CD4^{+} TIM3^{+} LAG3^{-} TIGIT^{+} PD1^{+}	*
LAG3/TIGIT/PD1	CD45^{+} CD3^{+} CD4^{+} TIM3^{-} LAG3^{+} TIGIT^{+} PD1^{+}	*
TIM3/LAG3/TIGIT/PD1	CD45^{+} CD3^{+} CD4^{+} TIM3^{+} LAG3^{+} TIGIT^{+} PD1^{+}	*
Total Exhausted	CD45^{+} CD3^{+} CD4^{+} plus positive for two or more of the following: TIM3, LAG3, TIGIT, PD1 (populations marked with an * above)	✓
**CD8^{+} T cells**		
Total	CD45^{+} CD3^{+} CD8^{+}	✓
Naïve	CD45^{+} CD3^{+} CD8^{+} CD45RA^{+} CCR7^{+}	✓

Central memory (TCM)	$CD45^+ CD3^+ CD8^+ CD45RA^- CCR7^+$	✓
Effector memory (TEM)	$CD45^+ CD3^+ CD8^+ CD45RA^- CCR7^-$	✓
Terminally differentiated effector memory (TEMRA)	$CD45^+ CD3^+ CD8^+ CD45RA^+ CCR7^-$	✓
**$CD8^+$ T cells expressing exhaustion markers**		
Non-exhausted	$CD45^+ CD3^+ CD8^+ TIM3^- LAG3^- TIGIT^- PD1^-$	✓
TIM3 only	$CD45^+ CD3^+ CD8^+ TIM3^+ LAG3^- TIGIT^- PD1^-$	
LAG3 only	$CD45^+ CD3^+ CD8^+ TIM3^- LAG3^+ TIGIT^- PD1^-$	
TIGIT only	$CD45^+ CD3^+ CD8^+ TIM3^- LAG3^- TIGIT^+ PD1^-$	
PD1 only	$CD45^+ CD3^+ CD8^+ TIM3^- LAG3^- TIGIT^- PD1^+$	
TIM3/LAG3	$CD45^+ CD3^+ CD8^+ TIM3^+ LAG3^+ TIGIT^- PD1^-$	*
TIM3/TIGIT	$CD45^+ CD3^+ CD8^+ TIM3^+ LAG3^- TIGIT^+ PD1^-$	*
TIM3/PD1	$CD45^+ CD3^+ CD8^+ TIM3^+ LAG3^- TIGIT^- PD1^+$	*
LAG3/TIGIT	$CD45^+ CD3^+ CD8^+ TIM3^- LAG3^+ TIGIT^+ PD1^-$	*
LAG3/PD1	$CD45^+ CD3^+ CD8^+ TIM3^- LAG3^+ TIGIT^- PD1^+$	*
TIGIT/PD1	$CD45^+ CD3^+ CD8^+ TIM3^- LAG3^- TIGIT^+ PD1^+$	*
TIM3/LAG3/TIGIT	$CD45^+ CD3^+ CD8^+ TIM3^+ LAG3^+ TIGIT^+ PD1^-$	*
TIM3/LAG3/PD1	$CD45^+ CD3^+ CD8^+ TIM3^+ LAG3^+ TIGIT^- PD1^+$	*
TIM3/TIGIT/PD1	$CD45^+ CD3^+ CD8^+ TIM3^+ LAG3^- TIGIT^+ PD1^+$	*
LAG3/TIGIT/PD1	$CD45^+ CD3^+ CD8^+ TIM3^- LAG3^+ TIGIT^+ PD1^+$	*
TIM3/LAG3/TIGIT/PD1	$CD45^+ CD3^+ CD8^+ TIM3^+ LAG3^+ TIGIT^+ PD1^+$	*
Total Exhausted	$CD45^+ CD3^+ CD8^+$ plus positive for two or more of the following: TIM3, LAG3, TIGIT, PD1 (populations marked with an * above)	✓
**B cells**		
Total	$CD45^+ CD19^+$	✓
Naïve	$CD45^+ CD19^+ CD27^- IgD^+$	✓
Memory	$CD45^+ CD19^+ CD19^+ CD27^+$	✓
Non-Class Switched Memory (NCSM)	$CD45^+ CD19^+ CD19^+ CD27^+ IgD^+$	✓
Class Switched Memory (CSM)	$CD45^+ CD19^+ CD19^+ CD27^+ IgD^-$	✓
**Other lymphocytes**		
Natural killer (NK) cells - Total	$CD45^+ CD3^- CD16/CD56^+$	✓
Proliferative NK cells	$CD45^+ CD3^- CD16/CD56^+ CD57^-$	✓
Less proliferative NK cells	$CD45^+ CD3^- CD16/CD56^+ CD57^+$	✓
Natural killer T (NKT) cells - Total	$CD45^+ CD3^+ CD16/CD56^+$	✓
Proliferative NKT cells	$CD45^+ CD3^+ CD16/CD56^+ CD57^-$	✓
Less proliferative NKT cells	$CD45^+ CD3^+ CD16/CD56^+ CD57^+$	✓
Large granular lymphocytes (LGL) with lower proliferative potential	$CD45^+ CD3^+ CD16/CD56^- CD57^+$	✓
