# Supplementary material for: Immunophenotyping of Paired Bone Marrow and Peripheral Blood: Implications for Monitoring Plasma Cell Disorders
Source: Cancers (Basel). 2026 Oct 23;18(21):3428. doi: 10.3390/cancers18213428 (PMC13761744; doi:10.3390/cancers18213428)
Supplement: Supplementary file 1 [file cancers-18-03428-s001.zip › Table S7 - clean vs bloody bm - percent gated.pdf]

Table S7. Comparison of immune profiles of paired bone marrow aspirate and peripheral blood of 60 patients, stratified by bone marrow cellularity, being evaluated for plasma cell disorders at Roswell Park Comprehensive Cancer Center (2020-2024)

	>10,500 BMA leukocytes/μL (n=23)			≤10,500 BMA leukocytes/μL (n=37)			Δρ
	ρ (95% CI)	Strength	p-value↕	ρ (95% CI)	Strength	p-value↕	Mdn (95% CI)‡
**Percent Gated** #							
Lymphocytes, total	.79 (.56 - .91)	Strong	.0002 *	.79 (.62 - .88)	Strong	<.0041 *	.01 (-.22, .24)
T cells, total	.62 (.28 - .82)	Moderate	.0011 *	.88 (.77 - .93)	Strong	<.0041 *	.25 (-.01, .62)
$\gamma\delta^+$ T cells	.98 (.94 - .99)	Very strong	.0002 *	.94 (.89 - .97)	Very strong	<.0041 *	-.03 (-.11, .04)
**$CD4^+$ T cells**							
Total	.90 (.77 - .96)	Strong	.0002 *	.81 (.65 - .90)	Strong	<.0041 *	-.08 (-.30, .12)
Naïve	.95 (.88 - .98)	Very strong	.0002 *	.91 (.83 - .95)	Very strong	<.0041 *	-.03 (-.17, .06)
TCM	.79 (.56 - .91)	Strong	.0002 *	.88 (.78 - .94)	Strong	<.0041 *	.09 (-.09, .33)
TEM	.76 (.51 - .89)	Strong	.0002 *	.81 (.66 - .90)	Strong	<.0041 *	.04 (-.22, .38)
TEMRA	.79 (.56 - .91)	Strong	.0002 *	.67 (.44 - .82)	Moderate	<.0041 *	-.12 (-.43, .20)
Treg	.72 (.44 - .87)	Strong	.0002 *	.69 (.47 - .83)	Moderate	<.0041 *	-.02 (-.31, .29)
Non-exhausted↑	.89 (.75 - .95)	Strong	.0002 *	.86 (.74 - .92)	Strong	<.0041 *	-.03 (-.20, .17)
Exhausted↓	.67 (.36 - .85)	Moderate	.0002 *	.84 (.71 - .92)	Strong	<.0041 *	.15 (-.08, .51)
**$CD8^+$ T cells**							
Total	.85 (.66 - .93)	Strong	.0002 *	.97 (.93 - .98)	Very strong	<.0041 *	.11 (-.01, .38)
Naïve	.83 (.63 - .92)	Strong	.0002 *	.90 (.82 - .95)	Very strong	<.0041 *	.07 (-.11, .35)
TCM	.56 (.19 - .79)	Moderate	.0040 *	.76 (.57 - .87)	Strong	<.0041 *	.17 (-.17, .62)
TEM	.72 (.43 - .87)	Strong	.0002 *	.81 (.66 - .90)	Strong	<.0041 *	.09 (-.17, .44)
TEMRA	.94 (.86 - .97)	Very strong	.0002 *	.93 (.87 - .96)	Very strong	<.0041 *	-.01 (-.11, .11)
Non-exhausted↑	.90 (.78 - .96)	Very strong	.0002 *	.95 (.90 - .97)	Very strong	<.0041 *	.04 (-.08, .25)
Exhausted↓	.80 (.59 - .91)	Strong	.0002 *	.89 (.80 - .94)	Strong	<.0041 *	.09 (-.12, .31)
**B cells**							
Total	.92 (.83 - .97)	Very strong	.0002 *	.89 (.80 - .94)	Strong	<.0041 *	-.02 (-.22, .12)
Naïve	.95 (.88 - .98)	Very strong	.0002 *	.94 (.89 - .97)	Very strong	<.0041 *	.00 (-.10, .09)
Memory	.69 (.39 - .86)	Moderate	.0002 *	.88 (.77 - .94)	Strong	<.0041 *	.17 (-.08, .51)
NCSM	.89 (.75 - .95)	Strong	.0002 *	.94 (.89 - .97)	Very strong	<.0041 *	.05 (-.06, .27)

CSM	.70 (.41 - .86)	Strong	.0002 *	.86 (.75 - .93)	Strong	<.0041 *	.16 (-.07, .45)
**NK/NKT cells**							
NK, total	.56 (.19 - .79)	Moderate	.0040 *	.90 (.82 - .95)	Very strong	<.0041 *	.32 (.00, .76) †
NK, $CD57^{+}$	.62 (.29 - .82)	Moderate	.0009 *	.89 (.79 - .94)	Strong	<.0041 *	.24 (.00, .60) †
NK, $CD57^{-}$	.61 (.26 - .82)	Moderate	.0014 *	.87 (.75 - .93)	Strong	<.0041 *	.25 (-.06, .67)
NKT, total	.94 (.86 - .97)	Very strong	.0002 *	.93 (.87 - .97)	Very strong	<.0041 *	-.00 (-.10, .10)
NKT, $CD57^{+}$	.93 (.83 - .97)	Very strong	.0002 *	.98 (.96 - .99)	Very strong	<.0041 *	.05 (-.01, .16)
NKT, $CD57^{-}$	.86 (.68 - .94)	Strong	.0002 *	.82 (.68 - .90)	Strong	<.0041 *	-.02 (-.24, .18)
LGL, $CD57^{+}$	.95 (.89 - .98)	Very strong	.0002 *	.94 (.88 - .97)	Very strong	<.0041 *	-.01 (-.13, .07)
**Ratios of Absolute Cell Counts**							
CD4/CD8	.91 (.79 - .96)	Very strong	.0002 *	.94 (.88 - .97)	Very strong	<.0041 *	.02 (-.07, .20)
$CD4^{+}$ Tregs/$CD8^{+}$ T cells	.84 (.65 - .93)	Strong	.0002 *	.91 (.83 - .95)	Very strong	<.0041 *	.06 (-.08, .36)
$CD4^{+}$ Naïve/memory ∫	.95 (.89 - .98)	Very strong	.0002 *	.96 (.92 - .98)	Very strong	<.0041 *	.01 (-.07, .10)
$CD4^{+}$ TEM/TCM	.81 (.60 - .92)	Strong	.0002 *	.78 (.62 - .88)	Strong	<.0041 *	-.03 (-.26, .26)
$CD4^{+}$ Exhausted/non-exh. ↓↑	.82 (.62 - .92)	Strong	.0002 *	.93 (.87 - .96)	Very strong	<.0041 *	.10 (-.03, .36)
$CD4^{+}$ TEM/Exhausted ↓	.90 (.78 - .96)	Very strong	.0002 *	.67 (.45 - .82)	Moderate	<.0041 *	-.20 (-.54, .07)
$CD8^{+}$ Naïve/memory ∫	.81 (.61 - .92)	Strong	.0002 *	.85 (.73 - .92)	Strong	<.0041 *	.03 (-.16, .29)
$CD8^{+}$ TEM/TCM	.40 (-.02 - .70)	Weak	.0539	.65 (.41 - .80)	Moderate	<.0041 *	.25 (-.22, .76)
$CD8^{+}$ Exhausted/non-exh. ↓↑	.89 (.76 - .95)	Strong	.0002 *	.91 (.83 - .95)	Very strong	<.0041 *	.03 (-.14, .19)
$CD8^{+}$ TEM/Exhausted ↓	.77 (.52 - .90)	Strong	.0002 *	.69 (.47 - .83)	Moderate	<.0041 *	-.08 (-.38, .25)
B cells Naïve/memory	.92 (.82 - .97)	Very strong	.0002 *	.95 (.91 - .98)	Very strong	<.0041 *	.04 (-.05, .17)

Symbols: # lymphocytes stated as a percent of total leukocytes. All other populations stated as a percent of total lymphocytes. ↕ p-values corrected for false discovery rate using the Benjamini-Hochberg procedure. ‡ median and 95% CI for difference (Δ) in ρ, from bootstrap-based test. * $p \leq .05$ after correction for false discovery rate. † Δρ is significantly different from zero. ↑ Non-exhausted: not expressing any of the following exhaustion markers: PD1, TIGIT, LAG3, TIM3. ↓ Exhausted: Expressing two or more of the following exhaustion markers: PD1, TIGIT, LAG3, TIM3. ∫ Memory=TEM+TCM. **Abbreviations:** BMA=bone marrow aspirate; CD=cluster of differentiation; CI=confidence interval; CSM= class-switched memory; Δ=difference; γδ=gamma-delta T cells; IQR=interquartile range; n=number; LGL=large granular lymphocytes; Mdn=median; NCSM=non-class-switched memory; NK=natural killer cells; NKT=natural killer T cells; PB=peripheral blood; ρ=Spearman correlation coefficient; TCM=central memory T cells; TEM=effector memory T cells; TEMRA=Terminally differentiated TEM (TEM re-expressing CD45RA)
